# Approaches to Refining Estimates of Global Burden and Economics of Dengue

**DOI:** 10.1371/journal.pntd.0003306

**Published:** 2014-11-20

**Authors:** Donald S. Shepard, Eduardo A. Undurraga, Miguel Betancourt-Cravioto, María G. Guzmán, Scott B. Halstead, Eva Harris, Rose Nani Mudin, Kristy O. Murray, Roberto Tapia-Conyer, Duane J. Gubler

**Affiliations:** 1 Schneider Institutes for Health Policy, Heller School, Brandeis University, Waltham, Massachusetts, United States of America; 2 Carlos Slim Health Institute, Mexico City, Mexico; 3 Pedro Kourí Tropical Medicine Institute, Havana, Cuba; 4 Dengue Vaccine Initiative, Rockville, Maryland, United States of America; 5 University of California, Berkeley, California, United States of America; 6 Ministry of Health, Putrajaya, Malaysia; 7 Baylor College of Medicine and Texas Children's Hospital, Houston, Texas, United States of America; 8 Duke-NUS Graduate Medical School, Singapore; Hospital for Tropical Diseases, Vietnam

## Abstract

Dengue presents a formidable and growing global economic and disease burden, with around half the world's population estimated to be at risk of infection. There is wide variation and substantial uncertainty in current estimates of dengue disease burden and, consequently, on economic burden estimates. Dengue disease varies across time, geography and persons affected. Variations in the transmission of four different viruses and interactions among vector density and host's immune status, age, pre-existing medical conditions, all contribute to the disease's complexity. This systematic review aims to identify and examine estimates of dengue disease burden and costs, discuss major sources of uncertainty, and suggest next steps to improve estimates. Economic analysis of dengue is mainly concerned with costs of illness, particularly in estimating total episodes of symptomatic dengue. However, national dengue disease reporting systems show a great diversity in design and implementation, hindering accurate global estimates of dengue episodes and country comparisons. A combination of immediate, short-, and long-term strategies could substantially improve estimates of disease and, consequently, of economic burden of dengue. Suggestions for immediate implementation include refining analysis of currently available data to adjust reported episodes and expanding data collection in empirical studies, such as documenting the number of ambulatory visits before and after hospitalization and including breakdowns by age. Short-term recommendations include merging multiple data sources, such as cohort and surveillance data to evaluate the accuracy of reporting rates (by health sector, treatment, severity, etc.), and using covariates to extrapolate dengue incidence to locations with no or limited reporting. Long-term efforts aim at strengthening capacity to document dengue transmission using serological methods to systematically analyze and relate to epidemiologic data. As promising tools for diagnosis, vaccination, vector control, and treatment are being developed, these recommended steps should improve objective, systematic measures of dengue burden to strengthen health policy decisions.

## Author Summary

Dengue is the most common mosquito-transmitted viral disease. It represents a formidable public health problem that is expanding in both infection rates and geographical range. Fortunately, vaccines, improved diagnostics, innovative vector control approaches, and other disease control methods are under development. Despite the importance of dengue, there is substantial uncertainty about the magnitude of the disease burden and economic cost of dengue, particularly in the number of symptomatic dengue infections. There is substantial variation in national reporting systems for dengue, which hinders accurate estimates of total cases and, therefore, of economic burden. Here we suggest a combination of immediate, short-term, and longer term strategies to address this knowledge gap. Immediate strategies include, for example, documenting the number of ambulatory visits before and after hospitalization. Short-term recommendations include merging multiple data sources, such as cohort and surveillance data to improve estimates of dengue incidence. Long-term efforts include increasing the collection and analysis of seroprevalence and economic data, use of enhanced surveillance (e.g., use of incentives to improve reporting, include private sector sentinel sites). Implementing these steps would give policy makers more reliable, systematic data for strengthening and refining policies about the application and financing of new technologies to control dengue.

## Introduction

Dengue presents a formidable global economic and disease burden with around half the world's population estimated to be at risk of infection [1], [2]. Dengue transmission has intensified in the past decades, with outbreaks increasing in frequency, magnitude, and countries involved [3], [4]. Dengue disease varies across time and age of persons affected. This complexity results from the transmission of four different viruses affected by vector density, the host's immune status, age, pre-existing medical conditions and other factors [5], [6]. The impact of dengue has been measured in terms of both monetary value and public health metrics, such as disability-adjusted life-years (DALYs) [7], [8]. Here we use the term “burden of dengue illness” to refer to the amount of clinically apparent disease and mortality imposed by dengue in a population. Economic burden has three main components: (i) costs of illness, estimated from the total symptomatic episodes multiplied by the average costs per episode [9], [10], (ii) costs of dengue prevention, surveillance, and control strategies [11], [12], and (iii) other impacts of dengue, usually harder to estimate, such as effects of dengue outbreaks on tourism [13], co-morbidities and complications associated with dengue virus (DENV) infection [14]–[16], or the effects of the seasonal clustering of dengue on health systems [17]. Accurate estimates of the economic and disease burden of dengue are critical to track health progress, assess program impact and results, and inform decisions about health policy, research, and health service priorities [7], [18]–[20]. However, estimates of dengue burden have substantial variability due to limitations in the availability, quality, and use of data.

As promising technologies for vaccination, vector control, and disease management are being developed, more reliable measures of dengue illness burden are needed to produce better data on the economic cost of dengue. This systematic review aims to identify and examine estimates of dengue burden and their main sources of uncertainty and to develop an agenda for immediate, short-term, and long-term strategies to improve these estimates. Our main focus in this article is on the costs of illness, particularly from the challenges to estimate the total episodes of symptomatic DENV infections.

## Materials and Methods

**Figure 1:**
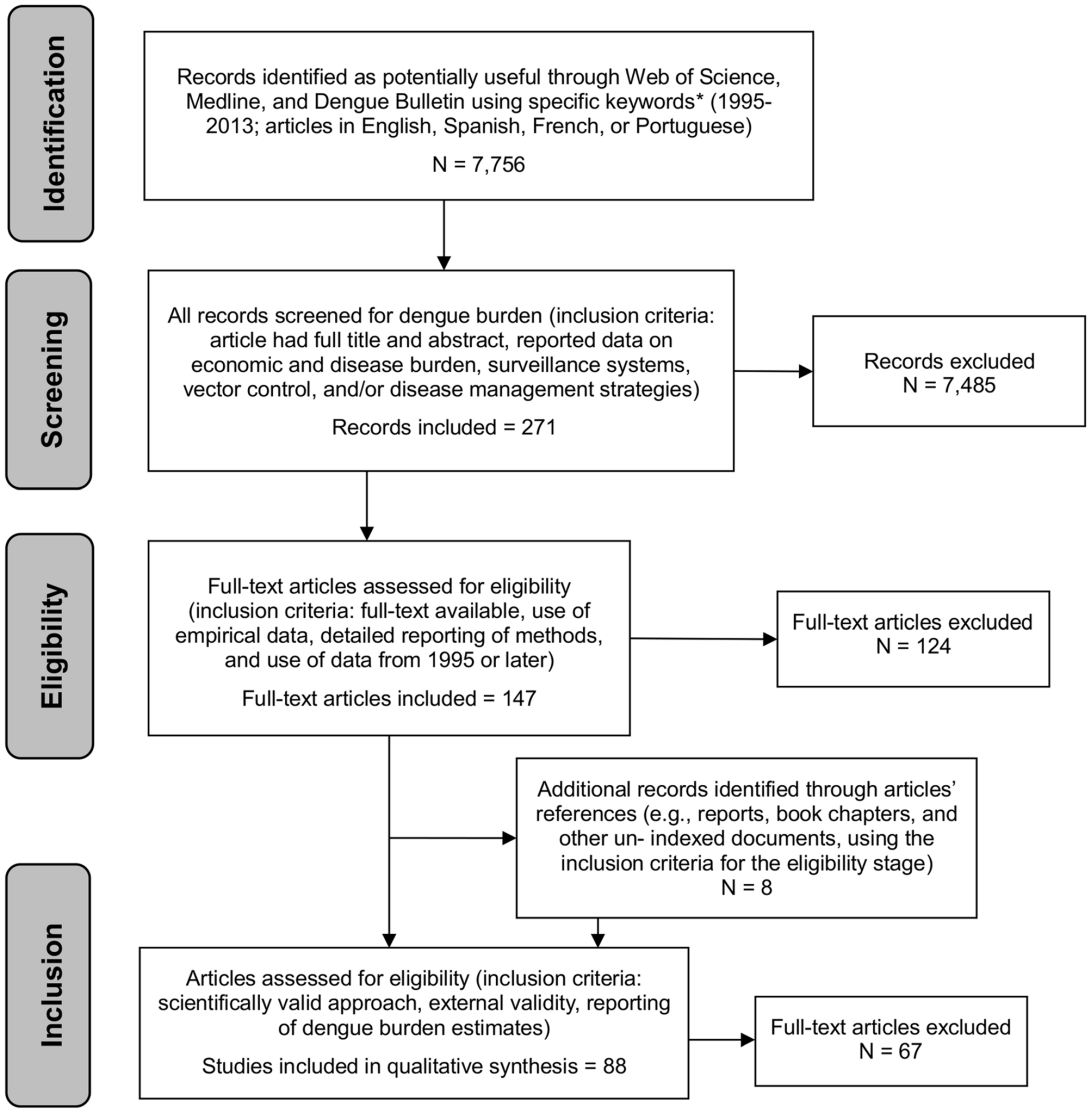
Review summary, PRISMA 2009 flow diagram. Notes: * Search includes articles published in the Web of Science, MEDLINE, or in WHO's *Dengue Bulletin* published between 1995 and 09/09/2013 in English, Spanish, French, or Portuguese, using the keyword “dengue” with the following list of keywords: surveillance, incidence, reporting, sensitivity, capture recapture, cohort, economics, costs, burden, *Aedes aegypti*, and control. Source: PRISMA flow diagram based on [22].

Available data on the economic and disease burden of dengue are limited. We conducted a systematic literature review of articles published or indexed in the Web of Science, MEDLINE, or in WHO's *Dengue Bulletin*, combining the keyword “dengue” with the following list of keywords: surveillance, incidence, reporting, sensitivity, capture-recapture, cohort, economics, costs, burden, *Aedes aegypti*, and control. In addition, we added findings from previous literature reviews on dengue disease and economic burden [9], [10], [21]. For relevance to current dengue surveillance and management, we included articles published from 1995 through 2013 in English, Spanish, French, or Portuguese. The inclusion criteria for articles at each step of the review process (i.e. identification, screening, eligibility, and inclusion) are shown in the PRISMA flow diagram [22] (Figure 1). The review process left us with 88 articles. Our goal was not to obtain numerical findings from the individual studies, but rather to summarize the main strategies and data used to estimate the economic and disease burden of dengue and the sources of variability in the burden estimates.

## Results

### Dengue burden data and sources of variability

Estimates of the disease and economic burden of dengue were derived by combining surveillance, clinical, and cost data. Since dengue is a reportable disease in many endemic countries, the incidence of dengue in a population can be estimated initially from cases reported to the surveillance system. But because surveillance systems are not designed to capture all episodes of symptomatic dengue, relatively low reporting rates lead to conservative incidence estimates [23]–[26]. Further, national dengue reporting systems show great diversity in design and implementation, and some developing countries have important resource limitations that hamper their ability to produce any systematic dengue-related data. Recent efforts to improve estimates of dengue burden include merging multiple data sources (e.g., health and surveillance data, private laboratories, experts' opinion) [27], [28], analyzing the relationship between cohort studies and routine reporting [25], [26], [29], and estimating incidence and/or reporting rates using covariates (e.g., healthcare access and quality, geographic and climate variables) [2], [8], [23].

To illustrate, there were about 2.2 million reported episodes of dengue illnesses to WHO in 2010, but estimates of total symptomatic dengue incidence vary widely. Bhatt and others [2] estimated 96.0 million dengue episodes globally. Their study combined a range of evidence of dengue transmission [1] with various sources of occurrence data (outbreak reports, cohort studies, online reporting, etc.), adjusting for the probability of occurrence of dengue based on socioeconomic, urban, and environmental covariates. Much of the dengue reporting occurs in areas of high transmission or during disease outbreaks, creating an upward trend in reports of dengue occurrence. Resulting models may have overstated total DENV infections. Also, the ratio of inapparent to apparent DENV infections varies substantially, depending on the age of patients, herd immunity and the circulating virus strain. The 2004 WHO global burden of disease (GBD) [30], estimated 9 million dengue episodes globally based on country-level datasets and information, and a systematic review of population-based incidence and mortality studies. By adjusting surveillance data with the rate of reporting of symptomatic DENV infections to health authorities, Shepard and others estimated about 30 million annual episodes treated in the medical system globally [31]. Last, Murray and others' GBD 2010 study [8] estimated global incidence of 0.2 million dengue episodes in 2010. Noting that their approach underestimated disease burden for dengue and other neglected tropical diseases; improved updates for 2013 are underway [32].

A review of studies on the economic burden of dengue in 2011 highlighted the relatively sparse literature and conflicting results of existing cost studies [33]. A recent report proposed procedures for costing dengue illness [19]. Extensions to that document that may help in refining dengue economic evaluations include estimating unit costs that are sensitive to productivity loss for workers that are not part of the formal economy (e.g., estimating the local marginal productivity of labor based on local wages averaged over the dengue season), examining local health-seeking behavior involving pharmacies or traditional healers, or using macro-costing techniques, which allow one to allocate overall operating costs among the outputs of a health facility [34] when person-level costs are unavailable. Estimates of health system congestion costs are also important; when health facilities are close to their capacity, the costs of an outbreak should also include costs that additional episodes impose on the system as a whole, like degradation of treatment quality of non-dengue patients [17].

Most important, improving current estimates of total dengue episodes is critical to quantify the disease and economic burden of dengue. Understanding the main sources of variability in the availability, quality, and use of reported data will allow for more comprehensive burden estimates. Consequently, through our literature review we identified the major sources of uncertainty, as a preliminary step in this direction.

#### Incompletely documented surveillance data

Many countries at risk to dengue transmission have no specific surveillance systems for dengue, in particular in the Western Pacific, South Asia, and Africa [1], [35]–[38]. Dengue competes with other public health and surveillance demands, making additional efforts to report dengue challenging. But dengue is clearly emerging as a major health problem [3], [4]. Without effective reporting systems, the burden of dengue cannot be accurately described geographically and quantitatively.

Surveillance systems in those countries reporting dengue illnesses are passive, dependent on an individual presenting to the healthcare system and the provider reporting the case to public health authorities. Passive surveillance systems are reasonably easy to implement, and make it possible to mobilize measures for epidemic control.

Underreporting of symptomatic DENV infections is the main source of uncertainty in burden of dengue estimates [9], [10]. DENV infections range from inapparent or mild febrile illness to severe or fatal hemorrhagic fever [4]. Inapparent infections also contribute to DENV virus transmission [39]. Most dengue episodes with mild symptoms and cases where the individual either does not seek treatment or visits private or alternative healthcare providers are not reported [27], [40], [41]. Recent evidence from Puerto Rico [42] also suggests underreporting of dengue deaths, in its comparably well-funded and effective surveillance system. Design and implementation limitations of dengue surveillance systems [41], [43]–[46], including insufficient feedback to reporting hospitals and health units, hinder national and global estimates of dengue disease burden and comparability across countries.

#### Variable dengue classification

Dengue classification can vary by region [4], [45]–[48]. As the epidemiology of dengue changed and new patterns of disease were observed, experts encountered problems with the WHO 1997 guidelines [4], [49] for classification of symptomatic DENV infections [50], [51]. Initially, dengue experts in some countries, such as India [52], Nicaragua [53], and Singapore [54], proposed or implemented new clinical categories and case definitions. This ultimately led to the new 2009 WHO Guidelines and case classification [4]. As use of the new 2009 WHO revised case classification expands globally, comparability between studies and countries over time may be affected if there is no overlap between old and new case definitions. But case definitions do not provide an economic description of patients. For the purpose of estimating economic dengue burden, documenting treatment setting (hospitalized and non-hospitalized) is essential to improve consistency and comparability of data.

#### Dissimilar reporting criteria

Evidence suggests that reporting rates vary by dengue severity, with better reporting for more severe episodes [55], [56]. Studies of reporting rates in the Americas and Southeast Asia have found that symptomatic dengue reporting rates are substantially higher among hospitalized cases than among ambulatory ones [10], [23]. The distribution between outpatient and inpatient treatment of symptomatic DENV infections is a substantial source of uncertainty in economic burden estimates of dengue. Often these proportions have been estimated only from expert opinion [27], [28], [57], [58]. The severity of dengue depends on age [39], with symptomatic cases occurring mostly in children in Asia and adults in the Americas [59], [60]. As dengue transmission expands in the Americas, the age distribution of disease expression will come to resemble that in Asia; conversely, as dengue transmission rates fall in Asia, disease expression will increasingly involve adults. Both primary and secondary DENV infections more frequently result in overt disease in older children and adults than in young children [59], [61]–[64].

Reporting of dengue can differ according to specific country-reporting policies, dengue severity, and type of treatment. For example, reporting of dengue episodes is primarily determined by hospitalization in some countries, including Thailand [45], Cambodia, and Viet Nam, [41]. Some countries, such as Cambodia [41], have limited the reporting of dengue episodes to those in children under 15 years of age. Furthermore, some countries or health facilities may avoid or minimize reporting of dengue or other illnesses due to concerns about tourism, government priorities, or domestic politics [13], [65].

#### Diverse diagnostic criteria

The degree of recognition of dengue symptoms may affect dengue reporting rates. For example, a recent study in Papua New Guinea suggested that clinicians were not aware of existing DENV infections, and most febrile illness were diagnosed as malaria [66]. In India, reporting to the central government is not mandatory, and recent research suggests low reporting and incomplete data. Recent estimates from a case study in Madurai, India, suggest that there are about 282 dengue episodes per each reported episode [67].

Variation in use of laboratory diagnostic tests may lead to variation in burden of dengue estimates. A small number of countries confirm reported dengue with lab tests [41], and in many of these countries, such as Mexico and Malaysia [27], [68], only a fraction of patients with undifferentiated fever are tested. One of the causes of under-reporting in hospitals – including well-funded health systems like Singapore and Puerto Rico – is related to under-diagnosis of dengue; due in part to the limited sensitivity of diagnostic tests or testing constraints based upon cost [69], [70].

#### Limited healthcare coverage

Limited healthcare coverage may impede the collection of accurate data. When there is limited access to primary healthcare, health facilities are remote from population centers, have limited operating hours, or require relatively high out-of-pocket payments, patients may opt to visit alternative healthcare providers, such as pharmacies or local healers (e.g., in Mexico [40] and India [67], respectively). People may also prefer homecare [40], or homeopathic treatments [71], making it difficult to estimate dengue incidence accurately.

#### Paucity of data from the private sector

Few private facilities and practitioners submit information on dengue cases, leading to substantial under-reporting of dengue episodes. Few studies have addressed the limited or absent data from the private sector in a systematic way. Based on data from private laboratory tests, a Malaysian study [27], [28] estimated that the reporting rate in the private sector was about 17%, compared with 34% in the public sector. Further, a prospective cohort study in Morelos, Mexico, found that 17% of dengue episodes were treated in the private sector, none of which were reported to the national surveillance system [40]. While reporting rates depend on the specific country, there is substantial under-reporting of dengue from the private sector and inadequate understanding of patients' health-seeking behavior and private health service utilization in general.

#### Underestimation of persistent symptoms

Current estimates of dengue burden commonly are based upon reporting of acute illnesses (1–7 days) and some studies extend further, totaling about 12 days [72]. However, recent studies in Singapore, Brazil, Peru, Sri Lanka, and Cuba suggest that dengue symptoms, usually including fatigue and depression, may affect some patient's quality of life for months [54], [73]–[78]. Economic and disease burden of dengue studies should at minimum include the febrile and convalescent phases of dengue, but ideally the overall duration of impaired quality of life [79]. If persistent symptoms of dengue are common, previous studies probably underestimate dengue burden by not including the full disease spectrum.

#### Variation in costing of dengue prevention and control

Few studies have examined the economic costs of dengue prevention and control activities comprehensively [12], [80]–[84]; and the methods are not standardized across studies. Costs of vector control have usually been estimated during dengue outbreaks [12], [81], [82], [84], except for a study in Puerto Rico [83], which examined dengue prevention and control costs across epidemic and non-epidemic years (2002–2007), or have focussed on targeted interventions, including community-based strategies, larviciding campaigns, and targeting productive breeding places [21]. Countries typically have a dengue prevention and control budget, but incur additional expenditures during disease outbreaks, which need to be acknowledged in economic burden studies.

#### Neglected economic impacts of dengue

Studies of the economic and disease burden of dengue have overlooked some economic impacts of dengue outbreaks, probably because data are sparse or non-existent, and there is too much uncertainty in the costing calculations. Despite anecdotal evidence that outbreaks of dengue reduces revenues from tourism [85], to our knowledge only one study has begun to quantify the potential economic impact of dengue outbreaks on tourism [13], and projected substantial economic losses from averted tourism. Dengue outbreaks also present substantial temporal and geographical clustering [86]–[89], which may result in a degradation of treatment quality or sub-optimal treatment decisions (e.g., diversion of severe episodes from hospitalization or speeding discharge), and delays in reporting and laboratory work. Most health systems cannot support the cost of maintaining service capacity in excess of expected demand [90]. Last, co-morbidities and complications associated with DENV infection [14]–[16] are another source of economic burden that needs to be considered.

### Refining burden estimates

The number of limitations in reporting symptomatic dengue infections makes it difficult to estimate the true burden of dengue illness, which is probably underestimated in most studies. In this section, we suggest immediate, short-, and long-term refinements in data collection and analysis to improve the accuracy of estimations of the total dengue episodes and other components of disease burden. Table 1 lists the main sources of variability in dengue burden estimates and possible ways to improve data collection, including a few examples for some suggested improvements [6], [70], [91]–[94]. In the remainder of this section, we discuss possible analysis refinements with currently available data, or at least, data that could be gathered in the short run with marginal additional efforts.

**Table 1:** Recommended refinements to improve estimates of dengue burden.

Limitation	Recommended refinement
Incompletely documented surveillance data	Prioritize quality over quantity: limit data collection to selected sites (including private sector); include laboratories as an active component of surveillance systems; provide incentives for accurate, complete, and timely data (e.g., systematic reminders to providers, services such as diagnostic testing); provide rapid and quality feedback of lab results to reporting hospitals and health units; make data available to public health authorities, policy makers, and health analysts. A good example of an enhanced surveillance system is the Sentinel Enhanced Dengue Surveillance System (SEDSS) in Puerto Rico [92].
	Use randomized stratified sampling procedures in selecting diverse surveillance sites (e.g. both ambulatory and hospitalized settings, and public, private, and other sectors, such as non-profits).
	Document how sentinel surveillance sites are chosen and define sampling criteria. Understand the representativeness of the data.
	Make dengue a notifiable disease in regions that have reported outbreaks or are at risk of infection.
	Define a minimum set of indicators for dengue surveillance systems, including dengue diagnosis, lab testing, reporting facility, sector (public or private), setting (hospitalized and non-hospitalized), and age.
	Assess the use of existing infrastructure for other diseases, such as laboratory and surveillance infrastructure for acute febrile illnesses such as influenza and enterovirus, or for malaria in Africa.
	Include time periods long enough to capture seasonal and epidemic fluctuations.
	Perform additional studies to expand routine surveillance: (i) Use school-based seroprevalence studies as an affordable basis for inferring infection rates, acknowledging the specificity, sensitivity, and cost of DENV diagnosis tests [6], [70]. (ii) Test anonymously to determine dengue prevalence in existing settings where blood samples are collected (e.g., clinical laboratories for diagnosing illness, screening settings as in maternity clinics or children's hospitals [94]), or use existing blood samples from national health surveys [91] when available. (iii) Incorporate questions about febrile illness into seroprevalence studies to clarify the relationship between DENV infection and apparent infection, which varies substantially across countries depending on the age of patients' herd immunity and circulating DENV strain. (iv) Improve methods to quantify dengue endemicity [93].
Variable dengue classification	Identify treatment setting for each dengue episode (hospitalized and non-hospitalized) to improve consistency and comparability of data, and to assess economic burden.
	Register the total number of visits for ambulatory and hospitalized patients (prior to and following hospitalization).
	Overlap new and old definitions to maintain comparability over time or create a crosswalk across definitions, since consistent definitions are necessary for comparison across countries and regions, but dengue definitions continue to evolve. One way to achieve this might be to operate both definitions in parallel for a sample of patients (e.g., in a few sentinel hospitals). Another possibility might be reviewing hospital records and reclassifying dengue episodes using the new criteria.
Dissimilar reporting criteria	Explicitly acknowledge and explain reporting criteria, and adjust for variation to make data comparable across countries.
	Adjust reporting rates by severity, with a reasonable approach being adjustments by type of treatment.
	Examine patterns of treatment in cohort or epidemiological studies to describe the distribution between hospitalized and non-hospitalized dengue episodes.
	Include breakdowns by age groups to improve the understanding of dengue epidemiology because severity depends on the age at onset of disease.
Diverse diagnostic criteria	To reduce costs, particularly during outbreaks, refer only a random sample of symptomatic patients to laboratories for dengue testing (e.g., Mexico [40]). Subsidize sampled patients to incentivize their testing and reporting.
	Combine laboratory results and reporting rates (from public and private sectors) to improve estimates of disease burden.
	Because limited familiarity with dengue is a constraint in areas recently affected by dengue, train healthcare providers (public and private) and use educational campaigns to increase awareness.
Limited healthcare coverage	To address underreporting in isolated areas, use mobile and community-based surveys of patients with febrile illness to improve understanding of health service utilization and dengue incidence.
Paucity of data from the private sector	Include public and private healthcare visits in cohort studies to improve understanding of patients' health-seeking behavior and private health service utilization.
	Combine data from treatment facilities with information from alternative sources, such as private laboratories, to estimate episodes in the private sector.
	Provide training, simplify data acquisition (e.g. integrated web-based systems), share reports, and generate incentives (as suggested elsewhere [41]) and penalties in the private sector to improve reporting (e.g., Singapore).
	Analyze private sector treatment costs, insurance, and out-of-pocket payments through financial or administrative hospital records, and household surveys.
Under-estimation of persistent symptoms	In studies of disease burden, include at least the acute and convalescent phases of dengue episodes.
	Include a follow-up of 90 days to one year on all or on a sample of study participants to ascertain severity, prevalence, reduction in quality of life of possible persistent symptoms of dengue, such as long-term fatigue and depression.
	Conduct additional research related to chronic dengue symptoms to improve the accuracy of disability weights.
Variation in costing of dengue prevention and control	Estimate prevention and vector control costs across epidemic and non-epidemic years.
	Identify all agencies and institutions involved in dengue prevention and control activities, specifying roles, activities performed, population covered. Include household prevention and control activities.
	Identify personnel, recurrent, and capital costs allocated to dengue control. Include costs of vector and disease surveillance, fumigation, larviciding, inspection, educational and awareness campaigns, clean-up and other activities.
Neglected impacts of dengue	Expand research studies: (i) Collaborate with major hospitals that treat dengue patients to examine impacts of dengue on hospital congestion and co-morbidities and complications associated with DENV infection. (ii) Collaborate with tourism and border agencies to compile data and examine the impact of dengue in tourism revenues.

#### Expansion factors

**Figure 2:**
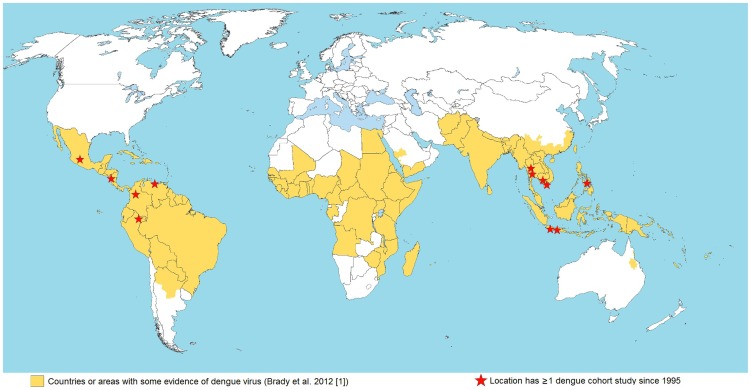
Countries and regions with evidence of dengue virus infections and cohort studies with published results since 1995. Notes: The map shows the approximate location of comprehensive cohort studies, based on a geographical area, that have examined dengue infections since 1995 for at least a year or a dengue season, although not all studies compare lab-confirmed dengue episodes with episodes reported to the surveillance system. In some locations (e.g., Kamphaeng Phet) there has been more than one cohort study. Sources: [1], [26], [40], [56], [89], [95], [96], [98]–[104].

Expansion factors (EF) are commonly used to adjust for underreporting of symptomatic DENV infections, and can be obtained from empirical studies and epidemiological surveillance. Data from a study sample can be extrapolated to the wider population, if assumptions are thought to remain consistent across time, space, and demographic groups. Empirical studies may be cohort studies [26], [40], [56], [89], [95]–[104], capture–recapture [24], [105], hospital prospective and retrospective studies [55], [106], [107], and national surveys [91]. Despite their importance as sources of high quality, reliable data, comprehensive cohort studies are limited in number (Figure 2), possibly because they are expensive, time-consuming, and not always feasible. Data from cohort studies also present challenges when extrapolating results to other regions, as these studies are usually done in areas of high-intensity dengue transmission, and reporting rates may vary in time and by region [26]. Other empirical study designs, such as capture-recapture studies or hospital prospective and retrospective studies, have proved very helpful in estimating disease burden, and further results could be achieved if combined systematically with other data. The identification of all febrile illnesses should be a common starting point for these studies.

When empirical data are not available, EFs might be obtained using a Delphi panel. A Delphi panel uses expert knowledge systematically, through several rounds of expert consultation with controlled opinion feedback, to help solve complex issues when data are insufficient. Nevertheless, the accuracy of estimates depends on the quality of available evidence and the knowledge of the constituent experts. This was illustrated in a recent study where a reporting rate of 26% [27], [28], obtained through a Delphi panel, was revised to 18% based on a multi-country equation using newer empirical data [23]. When an empirical study to estimate underreporting is not feasible, we recommend that Delphi panels include a diverse range of experts besides public health officials, particularly those in the treatment sector (e.g., healthcare centers, private physicians, or laboratories), and the use of empirical studies to advice assumptions where possible.

#### Analyzing covariates

Dengue epidemiology can vary substantially across regions and countries. Evidence suggests that dengue transmission is affected by factors such as geography, climate, time, demographics, income, urbanization, healthcare systems, mosquito population, herd immunity, and circulating DENV strain [29], [87], [89], [108]–[113]. Other factors, such as travel and trade, may also affect dengue transmission [3], [5], [6], [114]. These covariates may be used to adjust burden of dengue estimates through quantitative modeling, as illustrated by three recent publications [2], [8], [9].

A study by Murray and others estimated the global burden of dengue in terms of deaths and DALYs using a measure of accessibility to healthcare [8]. Similarly, Shepard et al. used a similar covariate to estimate the burden of dengue in Southeast Asia, but because dengue is primarily an urban disease, the authors focused on an index of healthcare quality [9], [23]. The underlying question was how to measure the idiosyncrasies of healthcare systems that lead to low reporting rates. Using a more holistic approach, Bhatt and others [2] recently examined the geographical distribution of dengue burden based on existing evidence of dengue transmission and adjusting their estimates with geographic, climate, socioeconomic, and urbanization covariates.

Each of these methods offers a way to refine estimates of dengue burden with currently available data. A promising strategy for future studies is to adopt a ‘portfolio’ approach, where a combination of strategies (empirical studies, expert opinion, and covariates) and diverse data sources are combined to overcome each source's limitations. For example, surveillance data may be refined using EFs obtained from cohort studies in specific regions, and the results extrapolated to a wider area through appropriate covariates, considering variation in EFs [26]. Recent studies have combined diverse data, including expert opinion, cohort and epidemiological studies, and climate, health, and socioeconomic covariates, to refine disease estimates [2], [27]. Another example of using existing data, finding patterns, and extrapolating to other countries is the WHO-CHOICE estimates [115], frequently used in studies of economic burden.

## Discussion

Multiple factors contribute to the variability in estimates of dengue burden, making it challenging to obtain accurate estimates. We recommend a series of strategies for improving dengue-burden estimates; however, some of them may be costly and therefore harder to achieve, and strategies themselves may need to be evaluated for their cost-effectiveness. Possibly the most important limitation has to do with limited availability, quality, and use of dengue surveillance data in many countries. New prospective studies to ascertain dengue burden better are needed, particularly in areas where reporting is least complete (or nonexistent), such as Africa or South Asia. However, several improvements in economic and disease burden estimates may be achieved with available data. Reported surveillance data should include a narrative about the system's main characteristics, including whether it includes the private sector, ambulatory episodes, cases of all ages, and type of lab confirmation, if any, of DENV infections reported. Most importantly, reporting to national surveillance systems should record each dengue episode as either hospitalized or ambulatory (i.e., never hospitalized). The use of covariates to estimate the burden of dengue can adjust for underreporting and/or to extrapolate to areas where there is no reporting at all [2], [8], [23]. It would be important to characterize the context for epidemiological dengue studies to describe why these studies were conducted at the specific time and place, and how those settings compare to others in the country or region. Understanding how specific variables affect the burden of dengue will help researchers improve burden estimates. The greatest source of uncertainty in existing burden of dengue studies comes from underreporting of symptomatic DENV infections, followed by the type of treatment of episodes. Probabilistic sensitivity analyses and tornado diagrams are helpful to understand the proportion of a confidence interval that arises from various sources of uncertainty [10], [40]. The biggest payoff for burden of dengue estimates would come from studies that can link and analyze existing data. For example, data from cohort studies and clinical trials could be re-analyzed and compared with officially reported dengue episodes to estimate EFs [104] and population-based economic burden. Understanding the health-seeking behavior of people with symptomatic DENV infections would, for example, allow researchers to estimate the probability that a dengue episode is reported as a function of setting (inpatient or outpatient), sector (public or private), case severity, age, type of facility, access to healthcare, and other variables in the surveillance system. We also expect that neglected impacts of dengue, such as decreases in tourism or health system congestion, would represent substantial costs during outbreaks.

We hope that future studies will obtain more accurate and comparable measures of economic and disease burden of dengue, for example, by documenting surveillance reporting criteria and adjustments used to estimate total symptomatic DENV infections (including adjustments for dengue episodes treated in the private sector or alternative health providers); using consistent case definitions; stratifying by treatment setting (hospitalized and non-hospitalized), severity, and age; using probabilistic sensitivity analysis to estimate uncertainty; and including comprehensive analysis of prevention and control costs. These improved estimates will be crucial for public health advisors and policy makers to identify optimal and cost-effective dengue control technologies and financing. Compared to other diseases with higher mortality rates or more frequent chronic symptoms, the DALY burden of dengue is relatively low; nevertheless, dengue poses a substantial burden on a large share of the world population. Estimates of dengue burden are sparse and there is significant room for refinement. Understanding the factors that shape the uncertainty around dengue burden and reporting will enable improvement of current estimates. Improving the methods to quantify dengue endemicity, for example, by using a measure of DENV incidence rather than disease, would also be a major improvement towards the goal of controlling dengue as it may allow more direct cross-country comparisons [93]. In the long run, we aim to identify the most cost-effective ways to control dengue, by combining various data sources and improving analytical tools. Costing studies can help us examine existing preventive and treatment approaches. Economic and epidemiological models can project costs and effectiveness of existing and alternative approaches in a range of settings.

Most likely the future paradigm of dengue prevention and control will require an integration of vaccine, vector control, and anti-viral strategies, and systematic, comparable measures of dengue burden will be increasingly important. Several organizations have called for the improvement of health data [18]. We, too, believe this is an essential global public good that will help prioritize and improve public health decisions locally and globally.

**Box 1. Learning points**

1. Dengue presents a formidable global economic and disease burden, but current estimates are probably conservative due to underreporting of dengue episodes and have substantial uncertainty, particularly in Africa and South Asia.

2. As promising technologies for vaccination, vector control, and disease management are being developed, objective systematic measures of dengue burden are needed to inform policies about their application and financing.

3. We propose immediate-, short-, and long-term strategies to improve current estimates of dengue burden, where the immediate approaches refine methods for analyzing existing data, especially from extending analysis of cohort studies.

4. Recommended short-term approaches entail merging multiple data sources, such as cohort and surveillance data, using expansion factors, and modeling dengue incidence using covariates to estimate reporting rates in more locations.

5. Promising long-term approaches include strengthening the capacity to collect, process, and analyze dengue data.

**Box 2. Key papers in the field of disease burden and economics**

1. Bhatt S, et al., 2013. The global distribution and burden of dengue. *Nature 496*: 504–507.

2. Shepard DS, et al., 2013. Economic and disease burden of dengue in Southeast Asia. *PLOS Negl. Trop. Dis*. 7: e2055.

3. Standish K, et al., 2010. High dengue case capture rate in four years of a cohort study in Nicaragua compared to national surveillance data. *PLOS Negl. Trop. Dis*. 4: e633.

4. Vong S, et al., 2012. Under-recognition and reporting of dengue in Cambodia: a capture-recapture analysis of the National Dengue Surveillance System. *Epidemiol. Infect. 140*: 491–499.

5. Wichmann O, et al. 2011. Dengue in Thailand and Cambodia: an assessment of the degree of under recognized disease burden based on reported cases. *PLOS Negl. Trop. Dis. 5*: e996.

## Supporting Information

Checklist S1 PRISMA 2009 checklist. Notes: NA denotes not applicable.
(DOCX)
